# Dietary sugar intake increases liver tumor incidence in female mice

**DOI:** 10.1038/srep22292

**Published:** 2016-02-29

**Authors:** Marin E. Healy, Sujoy Lahiri, Stefan R. Hargett, Jenny D.Y. Chow, Frances L. Byrne, David S. Breen, Brandon M. Kenwood, Evan P. Taddeo, Carolin Lackner, Stephen H. Caldwell, Kyle L. Hoehn

**Affiliations:** 1 Department of Pharmacology, University of Virginia, Charlottesville, VA, USA; 2 Department of Medicine, University of Virginia, Charlottesville, VA, USA; 3 Emily Couric Clinical Cancer Center, University of Virginia, Charlottesville, VA, USA; 4 School of Biotechnology and Biomolecular Sciences, University of New South Wales, Sydney, Australia; 5 Institute of Pathology, Medical University of Graz, Graz, Austria

## Abstract

Overnutrition can promote liver cancer in mice and humans that have liver damage caused by alcohol, viruses, or carcinogens. However, the mechanism linking diet to increased liver tumorigenesis remains unclear in the context of whether tumorigenesis is secondary to obesity, or whether nutrients like sugar or fat drive tumorigenesis independent of obesity. In male mice, liver tumor burden was recently found to correlate with sugar intake, independent of dietary fat intake and obesity. However, females are less susceptible to developing liver cancer than males, and it remains unclear how nutrition affects tumorigenesis in females. Herein, female mice were exposed to the liver carcinogen diethylnitrosamine (DEN) and fed diets with well-defined sugar and fat content. Mice fed diets with high sugar content had the greatest liver tumor incidence while dietary fat intake was not associated with tumorigenesis. Diet-induced postprandial hyperglycemia and fasting hyperinsulinemia significantly correlated with tumor incidence, while tumor incidence was not associated with obesity and obesity-related disorders including liver steatosis, glucose intolerance, or elevated serum levels of estrogen, ALT, and lipids. These results simplify the pathophysiology of diet-induced liver tumorigenesis by focusing attention on the role of sugar metabolism and reducing emphasis on the complex milieu associated with obesity.

Primary liver cancer is more than twice as prevalent in men than women^1^. The mortality of liver cancer is also sex-biased such that it is the second-leading cause of cancer-related death in men but the sixth-leading cause in women^2^. This sexual dimorphism in liver tumorigenesis is also observed in mice treated with the hepatocellular procarcinogen diethylnitrosamine (DEN) wherein male mice develop tumors at greater than twice the incidence of females^3^. Estrogen is one factor that has an important role in the sexual dimorphism of liver tumorigenesis as ovariectomy increases tumor incidence in female mice and estrogen treatment of male mice decreases tumorigenesis^4^. However, other factors including adiposity and hepatic fat content may also contribute to the sex discrepancy in tumorigenesis. For example, a large epidemiological study of more than 5 million adults in the UK showed that increasing body mass index is a very significant risk factor for liver cancer incidence in men, but not women^5^.

In a previous study we developed and tested a series of diets to investigate how altering dietary fat and sugar composition affected primary liver tumor incidence and burden in male mice. Because liver tumor incidence was very high in males (greater than 75%), it was not possible to distinguish diet-specific differences in incidence; however, overall tumor burden was strongly influenced by diet such that sugar intake was the major driver of tumor burden in male mice^6^. For example, two isocaloric diets were tested where the only difference was substituting part of the starch in a low-fat ‘normal chow’ diet with sucrose and fructose. This substitution was sufficient to increase tumor burden by seven-fold. In contrast, mice fed a ketogenic diet high in fat and low in sugar had the least tumor burden^6^. Importantly, obesity was completely uncoupled from tumorigenesis. Because of the sex discrepancy in liver tumor susceptibility, it is currently unknown whether female mice are also sensitive to the tumor-promoting effects of dietary sugar. Therefore, the goal of this study was to investigate the effects of dietary sugar and fat on liver tumorigenesis in DEN-treated female mice.

## Results

**Figure 1:**
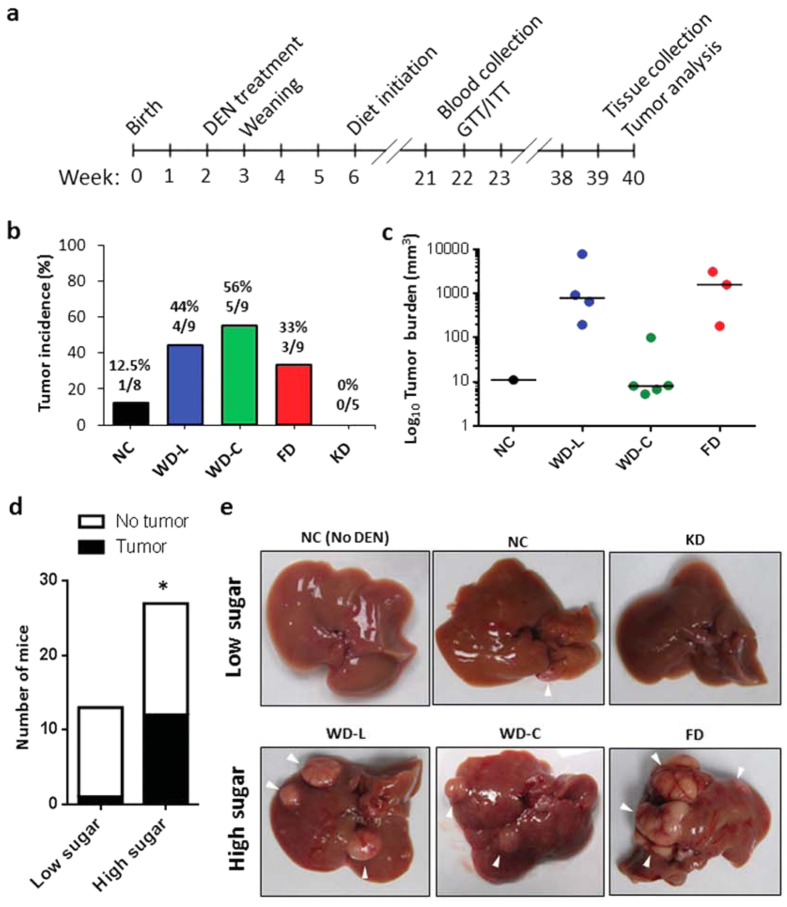
Dietary sugar is associated with increased tumor incidence in female C57BL/6N mice treated with DEN. (**a**) Diagram of study design. (**b**) Liver tumor incidence and (**c**) burden in 40-week-old mice treated with DEN at 2 weeks of age (n = 5–9). For (**c**), bars represent median tumor burden. **(d**) Liver tumor incidence grouped by dietary sugar consumption. *Low sugar* includes NC and KD; *high sugar* includes WD-L, WD-C and FD (analyzed using Fisher’s exact test, two-tailed). (**e**) Representative images of livers from each diet group. NC (No DEN) is shown as a control for normal liver.

The study design is illustrated in Fig. 1a. Female mice were treated with DEN at 14 days of age. Mice were weaned at 3 weeks of age and randomized to cages with non-littermates to avoid potential litter bias. From 6 weeks of age, mice were fed one of five diets with defined fat and sugar content. The five diets consisted of: a normal chow (NC) diet that was low in fat and sugar; a sucrose- and fructose-enriched diet (FD) that was low in fat; two high-sucrose Western diets (WD) containing either lard fat (WD-L) or coconut oil fat (WD-C); and a ketogenic diet (KD) that was high in lard fat and low in sugar (see *Methods* for precise composition). At 22 weeks of age (prior to tumor formation) mice were evaluated for diet effects on glucose and insulin tolerance, and blood samples were collected in the fed and fasted states for analysis of circulating nutrients and hormones. At 40 weeks of age mice were euthanized and evaluated for tumors. Liver tumor incidence was low (12.5%) in DEN-treated female mice fed NC diet, as expected. However, all three diets containing high sugar had increased tumor incidence such that mice fed WD-L had an incidence of 44%, mice fed WD-C had 56% incidence, and mice fed FD had 33% incidence. In contrast, mice fed the low-sugar ketogenic diet had no tumor incidence (Fig. 1b). Of the mice that developed tumors, those fed WD-L and FD had the highest median tumor burden (Fig. 1c). Mice that consumed high-sugar diets (WD-L, WD-C and FD) had significantly higher risk of liver tumor incidence compared to mice that consumed low-sugar diets (NC and KD) (Fig. 1d). Images of the livers with the median tumor burden for each group are shown in Fig. 1e and H&E-stained liver sections are shown in Fig. S1, which reveal extensive fat droplet accumulation in livers of the high-sugar fed mice.

**Figure 2:**
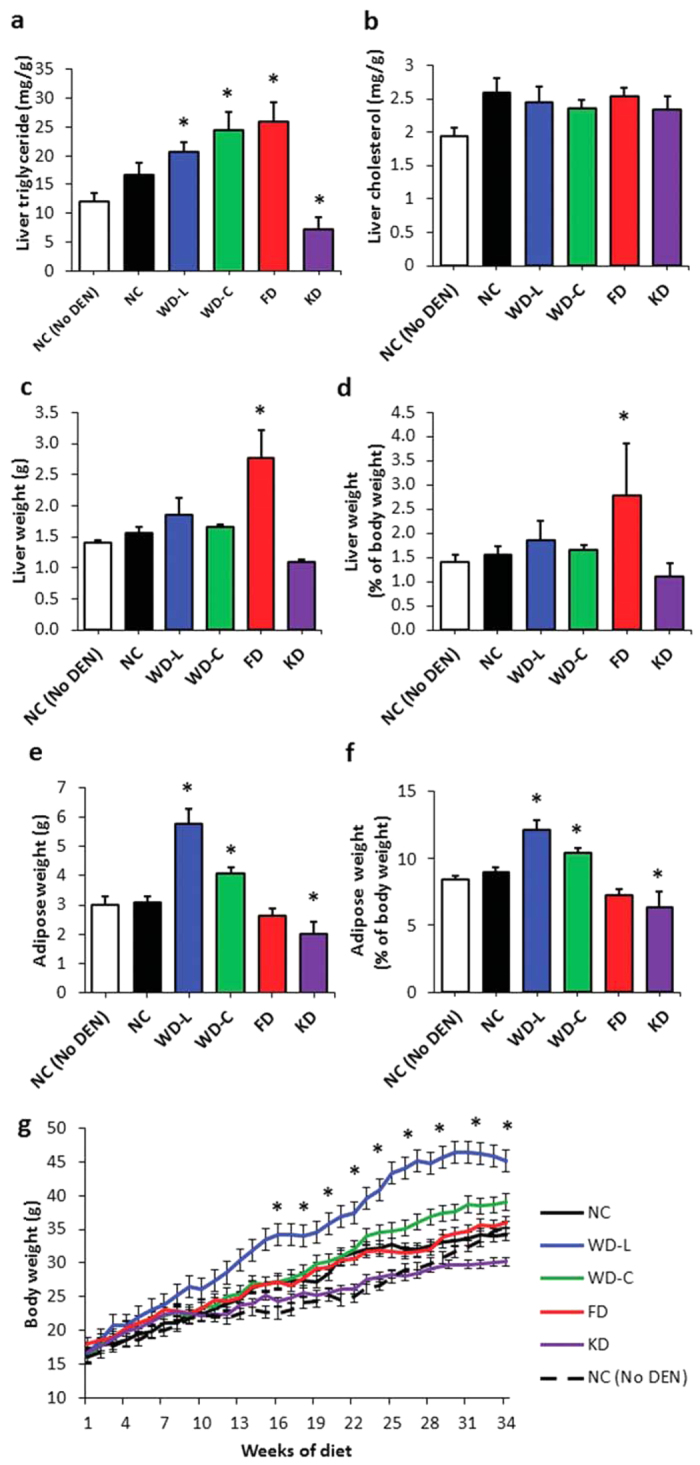
DEN and diet effects on hepatic lipid storage, adiposity, and body weight in female mice. Liver (**a**) triglyceride and (**b**) cholesterol content, (**c**) liver net weight and (**d**) as a percentage of body weight, (**e**) combined subcutaneous and gonadal adipose tissue weight, (**f**) adipose tissue weight as a percentage of body weight. (**a–f**) were determined from 40-week old mice at the end of the study period. (**g**) Mouse body weight changes during the 34 weeks of diet change. *indicates significant difference from NC, p < 0.05 (n = 5–9). Data are represented as mean ± SEM.

Since adiposity, hepatic lipid accumulation, and alterations in circulating lipids are associated with poor diet and liver cancer in humans and mice^3^,^5^,^6^,^7^,^8^, we evaluated the effect of each diet on these physiologic factors. Compared to DEN-treated mice fed NC diet, liver triglycerides were significantly higher in DEN-treated mice fed high-sugar diets (WD-L, WD-C and FD) and significantly lower in mice fed the high-fat low-sugar KD (Fig. 2a). In contrast, no significant differences in liver cholesterol levels were related to diet (Fig. 2b). Mice fed the high-sugar low-fat FD had significantly heavier livers compared to NC-fed controls (Fig. 2c,d). Mice fed the Western diets (WD-L and WD-C) had significantly higher adiposity than controls fed NC, whereas mice fed KD had significantly lower adiposity (Fig. 2e,f). Body weights of mice fed WD-L were significantly higher than controls fed NC from 15 weeks of diet through study completion, but body weight of mice fed other diets were not significantly different from normal chow (Fig. 2g).

Serum triglyceride levels were lower in the postprandial fed state of mice fed FD and higher in fasted serum of mice fed WD-C (Fig. S2a,b); however, no other diet groups had significant alterations in serum triglycerides compared to NC controls. Compared to NC controls, postprandial serum cholesterol levels were significantly elevated in the high-fat fed mice (WD-L, WD-C and KD), whereas only mice fed the high-fat high-sugar Western diets (WD-L and WD-C) had elevated serum cholesterol in the fasting state (Fig. S2c,d). Mice fed the high-fat low-sugar KD had significantly elevated free fatty acids in the postprandial state compared to NC controls. In the fasting state, however, there were no significant alterations in serum free fatty acids in any of the diet groups (Fig. S2e,f).

**Figure 3:**
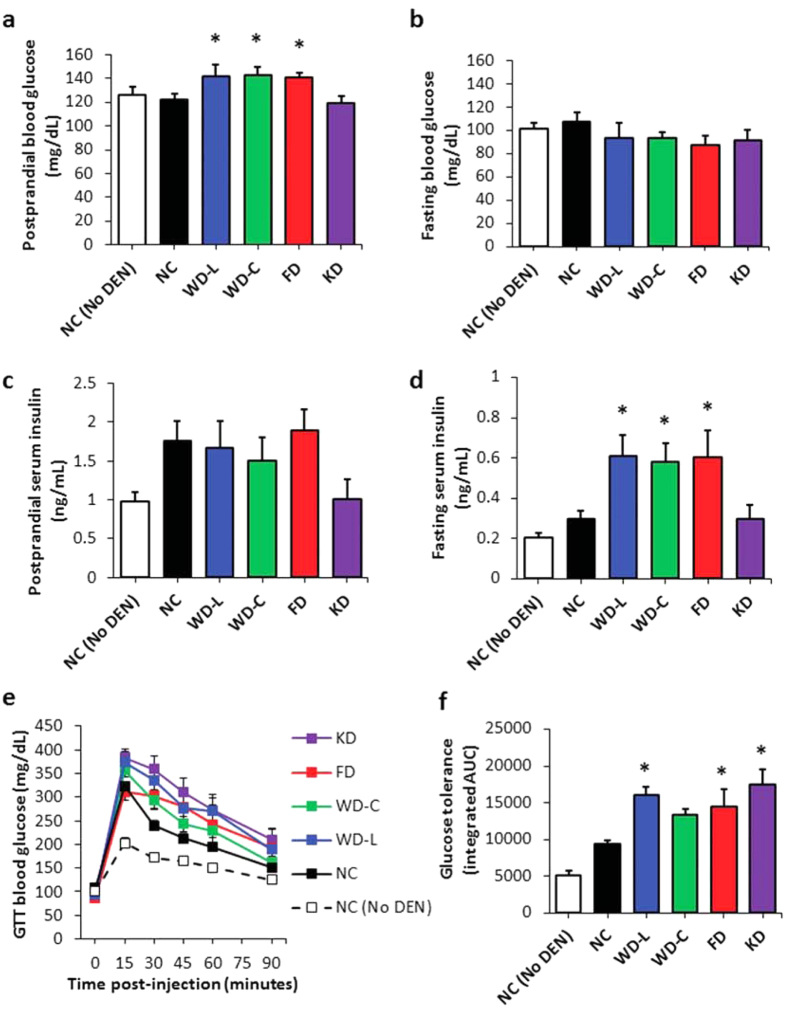
DEN and diet effects on fed and fasted glucose and insulin levels and glucose tolerance in 22-week old female mice. (**a**) Postprandial and (**b**) fasting blood glucose levels, and (**c**) postprandial and (**d**) fasting serum insulin levels. (**e**) Blood glucose levels in mice during a glucose tolerance test and (**f**) integrated area under the curve (AUC). *indicates significant difference from NC, p < 0.05 (n = 5–9). Data are represented as mean ± SEM.

Because hyperglycemia, hyperinsulinemia, and glucose and insulin intolerance have been described as independent risk factors for primary liver cancer incidence in humans^9^,^10^,^11^,^12^,^13^,^14^,^15^, we next examined the effects of each diet on whole body glucose metabolism in DEN-treated female mice. Mice fed high-sugar diets (WD-L, WD-C and FD) had significant increases in postprandial hyperglycemia compared to mice fed NC (Fig. 3a). In contrast, fasting blood glucose levels were not statistically altered in any diet group (Fig. 3b). Postprandial serum insulin levels were not statistically different from NC controls in any diet group (Fig. 3c); however, fasting serum insulin levels were significantly higher in mice fed high-sugar diets (WD-L, WD-C and FD) compared to controls fed NC (Fig. 3d). Finally, we tested the effect of diet on whole body glucose disposal and insulin sensitivity by subjecting mice to a glucose tolerance test (GTT) and insulin tolerance test (ITT). DEN-treated mice fed WD-L, FD and KD had significantly worse glucose tolerance than NC controls, and mice fed WD-C had a trend for impaired glucose tolerance (Fig. 3e,f). Mice fed WD-C had better glucose tolerance than WD-L, as expected^6^,^16^ (Fig. 3e,f). In contrast, the diets did not have deleterious effects on insulin tolerance, and in fact mice fed WD-C had improved insulin tolerance compared to NC controls (Fig. S2g,h).

Other serum markers associated with liver tumorigenesis were also investigated including estrogen (17β-estradiol) and alanine aminotransferase (ALT). Estrogen protects mice from liver tumor development in the DEN model^4^, and it contributes to the sexual dimorphism of liver cancer. However, in this study serum estrogen levels were not affected by DEN or diet (Fig. S2i). ALT is a marker of hepatocyte stress which is associated with increased risk of HCC development in humans^17^. However, only the KD group had significantly higher ALT compared to NC controls despite no tumor incidence in this group (Fig. S2j). Furthermore, DEN treatment did not affect ALT levels as there was no difference between DEN-naïve and DEN-treated mice fed NC.

**Figure 4:**
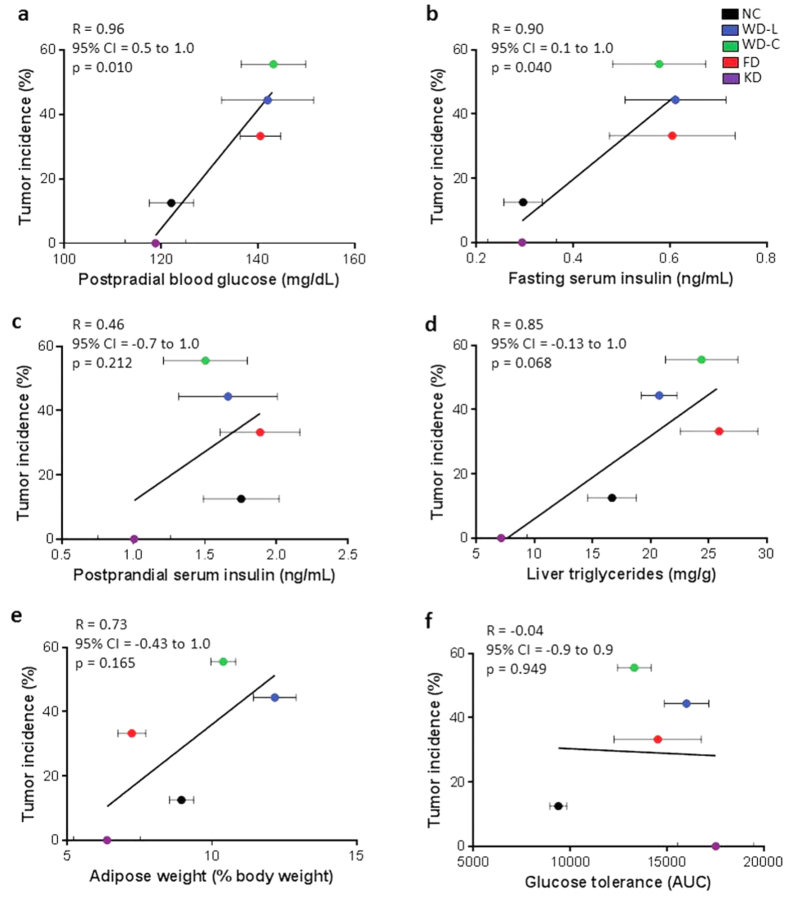
Correlation analysis between tumor incidence and metabolic risk factors in DEN-treated female mice. Correlations between tumor incidence and (**a**) postprandial blood glucose, (**b**) postprandial and (**c**) fasting serum insulin, (**d**) liver triglycerides, (**e**) adiposity, and (**f**) glucose tolerance area under the curve (AUC). Linear regression and Pearson’s correlation analyses were used to test for correlations with tumor incidence. Data are represented as mean ± SEM (n = 5–9).

We next determined correlations between tumor incidence and diet-related phenotypes (Fig. 4 and Fig. S3). Only postprandial blood glucose and fasting serum insulin levels were positively correlated with tumor incidence (Fig. 4a,b), while other known risk factors for liver cancer including liver triglyceride and cholesterol content, adiposity, serum lipids (*i.e.* triglycerides, cholesterol and free fatty acids), liver weight, postprandial serum insulin, glucose and insulin tolerance, and serum estrogen and ALT were not significantly correlated with tumor incidence (Fig. 4c–f, Figs S3a–h, and S4a–c).

## Discussion

The relationship between obesity and liver cancer is clearly evident in epidemiological studies in humans and dietary studies in mice^3^,^7^,^18^. However, since obesity is associated with a spectrum of metabolic disorders including hyperglycemia, fatty liver disease and others, it has remained difficult to determine whether obesity or one of its co-morbidities contributes to the pathogenesis of primary liver cancer. Most studies investigating the role of over-nutrition in liver cancer have used the Western diet containing 60% lard and sugar (*i.e.* sucrose and fructose) to drive tumorigenesis^3^,^7^. Although these Western diets cause both obesity and increased liver tumor burden, these experiments do not prove that obesity is the driver of liver tumorigenesis or progression. In a recent study, we tested a series of well-defined diets in male mice and observed that dietary sugar intake was sufficient to increase tumor burden without causing obesity^6^. The present study used female mice because they have a lower incidence of tumors compared to males, thereby enabling determination of differences in tumor incidence. Furthermore, female mice are more resistant to obesity-related liver cancer than males and it was unclear whether females would be sensitive to the tumor-promoting effects of dietary sugar.

The diets used in this study were identical to those previously tested in male mice^6^. The NC diet served as a baseline control for a low-fat low-sugar diet, while the WD-L diet containing 45% lard and sugar served as a positive control for obesity and liver tumorigenesis. The FD was used to investigate how increasing sugar content without altering fat affected obesity and tumorigenesis, while the KD increased fat content while keeping total carbohydrate levels very low. Finally, the WD-C diet contained 45% coconut oil instead of lard. This WD-C diet generally causes less glucose intolerance but more liver triglyceride accumulation^6^,^16^, thereby enabling the uncoupling of these parameters to determine whether the type of dietary fat could affect tumorigenesis.

Female mice that consumed diets rich in sugar had higher tumor incidence rates of approximately 3–5 times greater than mice fed normal chow, while no tumors were observed in female mice fed low sugar KD. Contingency analysis between sugar intake and tumor incidence clearly showed that female mice are highly susceptible to the tumor-promoting effects of dietary sugar despite having a lower overall incidence of liver tumorigenesis than males. The diet comparison that best illustrates the impact of sugar on tumor incidence is the contrast between NC and FD diets. These diets had identical nutrient composition with the exception of the source of carbohydrates. The carbohydrate load of the FD contained sucrose, fructose and corn starch, while the NC diet was comprised mainly of corn starch. The increase in tumor incidence caused by FD-feeding was completely independent of changes in adiposity. These data are consistent with other reports showing that fructose feeding increases precancerous hyperproliferative lesions and liver tumor incidence in carcinogen-treated rats^19^,^20^.

The sexual dimorphism in liver cancer may also be related to differences in insulin sensitivity where females are more protected from diet-induced insulin resistance than males. We therefore focused on the metabolic perturbations in glucose metabolism and their relationship to tumorigenesis. Our data showed that postprandial hyperglycemia was the factor most closely associated with liver tumor incidence in female mice. These data are consistent with several human epidemiological studies that have identified an association between HCC risk and dietary glycemic load^9^,^10^ or sugar consumption^11^. Elevated blood glucose levels are also associated with higher risk of HCC recurrence after curative treatment^12^. Furthermore, the anti-hyperglycemic agent metformin prevents DEN-induced liver tumorigenesis^21^. Thus, one possible mechanism linking dietary sugar to liver tumor incidence in this study may be directly related to elevations in blood glucose concentrations. A common characteristic of tumor cells is a heavy reliance on sugar metabolism for both energy production and biosynthesis to support proliferation^22^, thus it is plausible that high levels of circulating sugars are readily accessible by the liver and used as metabolic substrates to promote tumor development.

Elevated blood glucose stimulates the production of insulin, which is a strong stimulator of cell survival and proliferation and is associated with fatal liver cancer^13^; therefore, insulin represents a potential indirect mechanism for increased tumor caused by dietary sugar consumption. However, it should be noted that previous studies in insulin-deficient Akita mice have demonstrated that the lack of insulin resulted in increased liver tumorigenesis^23^, thereby suggesting that hyperglycemia rather than hyperinsulinemia is the most likely driving force for liver tumorigenesis. In the current study, fasting hyperinsulinemia was observed only in the mice fed the high-sugar diets (WD-L, WD-C and FD) and was significantly correlated with tumor incidence. Despite elevations in fasting serum insulin, fasting blood glucose was maintained at normal levels in these mice, indicating a compensatory increase in insulin levels was necessary to maintain blood glucose levels in a normal range. In the postprandial state there were no significant alterations in serum insulin levels despite elevated blood glucose levels in the mice fed high-sugar diets, suggesting that insulin secretion was not adequate to maintain normal blood glucose levels. Insulin resistance and glucose intolerance can result in the development of hyperglycemia and hyperinsulinemia and are associated with increased HCC risk^15^,^24^,^25^. However, in this study tumor incidence was not correlated with impaired glucose tolerance or insulin sensitivity determined by acute tests with bolus injections of glucose or insulin.

The two Western diets used in this study differed only in their source of fat, such that the WD-L was composed of 45% lard fat by kCal while the WD-C contained 45% coconut oil fat by kCal. Lard is composed primarily of long-chain fatty acids, whereas coconut oil is composed primarily of medium-chain fatty acids. Compared to long-chain fatty acids, medium-chain fatty acids are associated with better whole-body glucose tolerance despite greater hepatic steatosis^6^,^16^. Use of these diets allowed us to directly compare the effects of these specific types of fat on physiology, metabolism, and tumor development. Female mice fed WD-C were protected from whole-body glucose intolerance and had better insulin sensitivity compared to mice fed WD-L, similar to the effects observed in male mice^6^. However, a major unexpected difference was the lower tumor burden in female mice fed WD-C compared to WD-L because male mice fed WD-C had the greatest tumor burden of all diets^6^. This sex-specific effect of coconut oil in facilitating tumor burden in males while reducing tumorigenesis in females is difficult to reconcile, but one possible explanation is that estrogen promotes fatty acid oxidation through increasing expression of proteins involved in mitochondrial β-oxidation^26^. Compared to long chain fatty acids in lard, medium chain fatty acids in coconut oil do not require a transporter to enter mitochondria and therefore may have a higher tendency to be oxidized rather than esterified and stored^27^. Thus, females may be better primed to handle high mitochondrial flux of medium chain fatty acids compared to males. This is potentially supported by the observation that female mice fed WD-C had lower adiposity than those fed WD-L, whereas male mice fed WD-C had greater adiposity than those fed WD-L. Although coconut oil did not protect from tumor incidence, mice fed this diet generally had small tumors. Further work is needed to examine the mechanisms underlying this sex-specific response to different types of dietary fat.

The sexual dimorphism in liver cancer has been at least partially attributed to estrogen, as ovariectomy of female mice increases DEN-induced tumor development and estrogen administration restores protection^4^. Although dietary fat and carbohydrate content has been reported to influence circulating estrogen levels^28^, we did not observe any diet-induced effects on estrogen levels in the current study. However, one caveat of these data is that the mice were not synchronized for the same phase of estrous cycle at the time of serum collection therefore caution is required when interpreting these results.

The evolution of HCC usually involves a progression through fatty liver, nonalcoholic steatohepatitis (NASH), and tumorigenesis^29^,^30^. Fatty liver is sexually dimorphic in a similar pattern as liver cancer, such that fatty liver is less prevalent in women compared to men^31^. Similarly, triglyceride levels in the livers of the female mice in this study were approximately 50% less than male mice^6^. This sexual dimorphism of fatty liver in humans and mice corresponds with the stimulatory effects of estrogen on fatty acid oxidation; therefore, it is possible that the mechanisms of protection by estrogen may involve reduced hepatic fat accumulation. Although liver triglyceride levels did not significantly correlate with tumor incidence in the current study, high-sugar feeding caused significantly higher liver triglyceride levels and therefore the potential involvement of fatty liver in liver tumor initiation cannot be ruled out.

Alanine aminotransferase (ALT) is associated with liver tumor incidence in humans^32^ and in male DEN-treated mice^33^. In the current study serum ALT was significantly elevated only in the mice fed KD. This result is consistent with previous reports that prolonged ketogenic diet-feeding increases serum ALT in mice^34^. Serum ALT was not associated with liver tumorigenesis because mice fed KD had elevated ALT but no incidence of liver tumor. Furthermore, ALT was not induced by DEN treatment in female mice, which is attributed to the protective effects of estrogen^33^. Collectively, these data indicate that serum ALT is a poor biomarker of liver tumorigenesis in female mice.

Taken together, this study demonstrates that female mice are susceptible to the tumor-promoting effects of dietary sugar. Correlation analyses revealed that sugar-induced hyperglycemia and hyperinsulinemia were stronger risk factors for liver tumor incidence than excess adiposity, fatty liver, or glucose intolerance. Since nutrition is a modifiable environmental risk factor, these results suggest that restriction of dietary sugar may be an effective and feasible strategy to decrease the risk of developing primary liver cancer in humans. Additional epidemiological data and clinical trials will be needed to evaluate the relationship between dietary sugar consumption and liver tumor incidence in both men and women to determine whether sugar withdrawal can stall or reverse the course of liver tumor progression.

## Methods

### Mice breeding, DEN treatment, and diets

Male and female C57BL/6NHsd mice were purchased from Harlan Laboratories. Mice were housed in a temperature-controlled room (22 °C) on a 12-hour light/dark cycle in filter-top cages and with *ad libitum* access to food and water. Mice were time-mated to produce litters simultaneously. Offspring female mice were treated with DEN (25 mg/kg) at 14 days of age via intraperitoneal (*i.p.*) injection. Mice were weaned at 21 days of age and randomly allocated to cages with non-littermates of the same age. All weaned mice were fed a standard chow diet until 6 weeks of age. From 6 weeks of age, mice were fed *ad libitum* with one of five experimental diets that were prepared in-house as previously described^6^,^35^. Dietary changes were introduced one month after carcinogen treatment to avoid diet effects on initial carcinogen-mediated hepatocyte damage. All diets were identical as far as mineral, vitamin, choline, methionine, and gelatin content. Normal chow (NC) diet contained (w/w): 6% wheat bran, 67% uncooked corn starch, 17% casein, and 3% safflower oil. High sugar and fructose diet (FD) contained (w/w): 6% wheat bran, 21% uncooked corn starch, 31% sucrose, 15% fructose, 17% casein, and 3% safflower oil. Western diets (WD) contained (w/w): 18% uncooked corn starch, 5% wheat bran, 21% sucrose, 18% casein, 3% safflower oil, and either 23% lard (WD-L) or coconut oil (WD-C). High fat low sugar ketogenic diet (KD) contained (w/w): 71% lard, 16% casein, and 6% safflower oil. All animal experimental protocols were approved by the University of Virginia Animal Care and Use Committee. All animal research was carried out in accordance with the approved guidelines.

### Phenotyping

Body weights of mice were measured weekly from the time of diet initiation to study completion at 34 weeks of diet. Glucose tolerance and insulin tolerance testing was performed in mice at 22 weeks of age (16 weeks of experimental diet). For the glucose tolerance test (GTT), basal blood glucose was measured after a 12-hour fast, then mice were *i.p.* injected with a bolus of 25% glucose solution in saline (1.5 g/kg) and blood glucose levels were measured over time. For the insulin tolerance test (ITT), basal blood glucose was measured in the postprandial state, then mice were i.p. injected with a bolus of 0.125 U/mL insulin solution in saline (1 U/kg) and blood glucose levels were measured over time. Post-prandial glucose was measured in mice at 22 weeks of age in the random-fed condition at 09:00 hours. Fasting blood glucose was measured in mice at 22 weeks of age after a 12-hour overnight fast. Blood glucose levels were measured from the tail vein using an Accu-chek glucometer (Roche). Liver and serum lipid content was determined as described^36^ using colorimetric assay kits for triglycerides (Pointe Scientific), cholesterol (Infinity, Thermo Scientific), and free fatty acids (Cell Biolabs) according to the manufacturers’ protocols. Serum Alanine Aminotransferase was measured using an enzyme activity assay (Abnova) and serum 17β-estradiol was measured by ELISA (Cayman Chemical) according to the manufacturers’ protocols.

### Tissue collection and tumor analysis

Mice were euthanized at 40 weeks of age (34 weeks of experiment diet) in the random-fed state between 09:00 and 11:00 hours. Tumor incidence represents the percentage of mice in each group with visible surface-hemorrhaging liver tumors (≥0.5 mm in diameter). Liver tumor burden represents the total tumor volume per mouse. Tumor diameter was measured and used to calculate volume using the following formula: 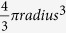. Liver tissue was snap-frozen in liquid nitrogen and stored at −80 °C until biochemical analysis. The weights of both gonadal and subcutaneous fat pads were summed and represented as combined adipose weight per animal.

### Statistical analysis

Group mean data were analyzed using one-way ANOVA and Fisher’s PLSD. Scatter plots were analyzed by linear regression to determine the line of best-fit, followed by Pearson’s correlation analysis to measure the correlation coefficient between two variables. Statistical significance was accepted at p < 0.05. Statistical analyses were performed using GraphPad Prism v6.00.

## Additional Information

**How to cite this article**: Healy, M. E. *et al.* Dietary sugar intake increases liver tumor incidence in female mice. *Sci. Rep.* **6**, 22292; doi: 10.1038/srep22292 (2016).

## Supplementary Material

Supplementary Information
