# Supplementary material for: Dietary sugar intake increases liver tumor incidence in female mice
Source: Sci Rep. 2016 Feb 29;6:22292. doi: 10.1038/srep22292 (PMC4770276; doi:10.1038/srep22292)
Supplement: Supplementary Information [file srep22292-s1.pdf]

Supplemental Data File

Dietary sugar intake increases liver tumor incidence in female mice

Marin E. Healy[1], Sujoy Lahiri[1], Stefan R. Hargett[1], Jenny D.Y. Chow[1], Frances L. Byrne[1,4], David S. Breen[1],

Brandon M. Kenwood[1], Evan P. Taddeo[1], Carolin Lackner[5], Stephen H. Caldwell[2,3], and Kyle L. Hoehn[1,2,3,4,*]

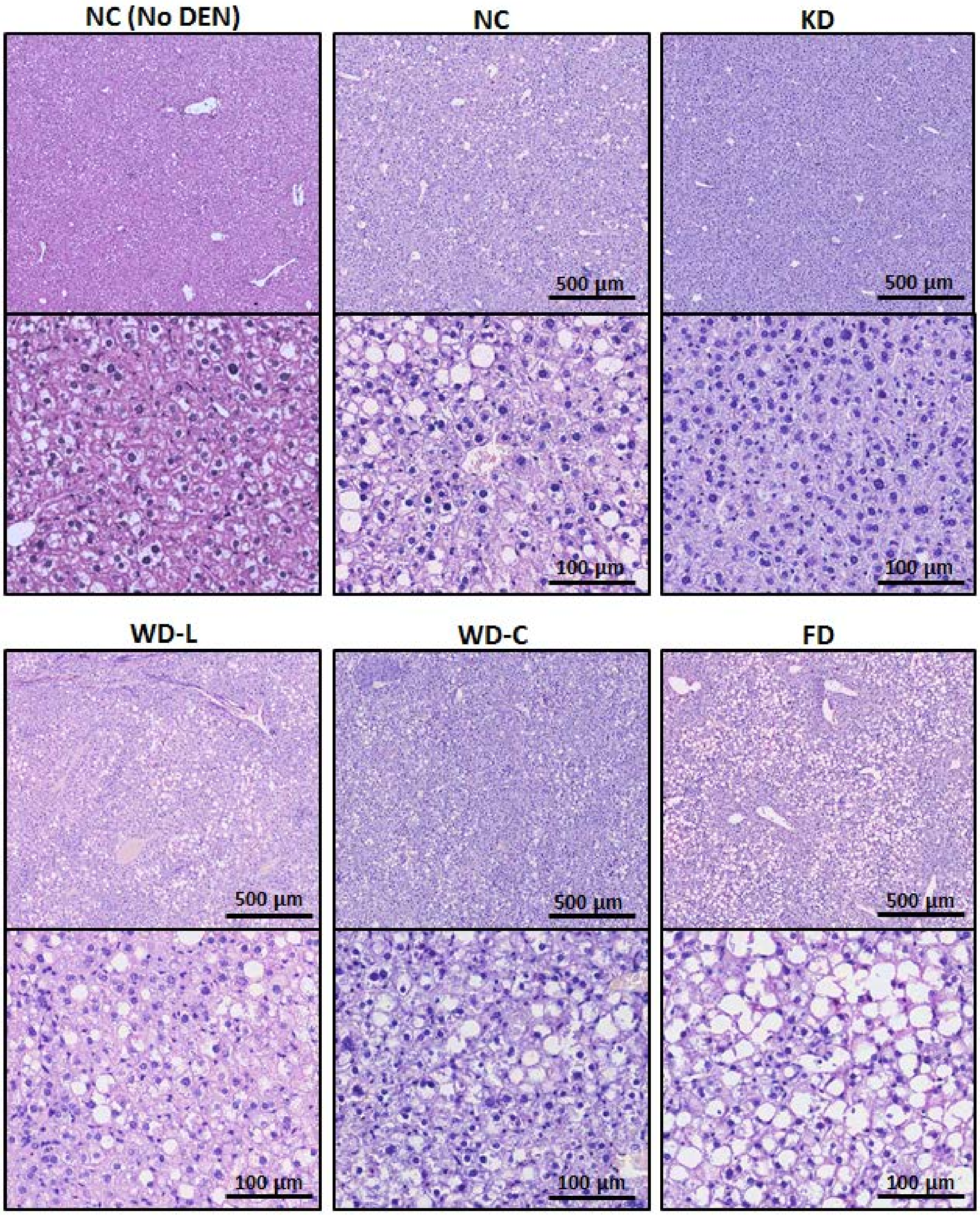

Figure S1. Liver histology of 40-week-old mice treated with DEN at 2 weeks of age. Representative images of H&E-stained liver sections from mice fed each diet. NC (No DEN) is shown as a control for normal liver.

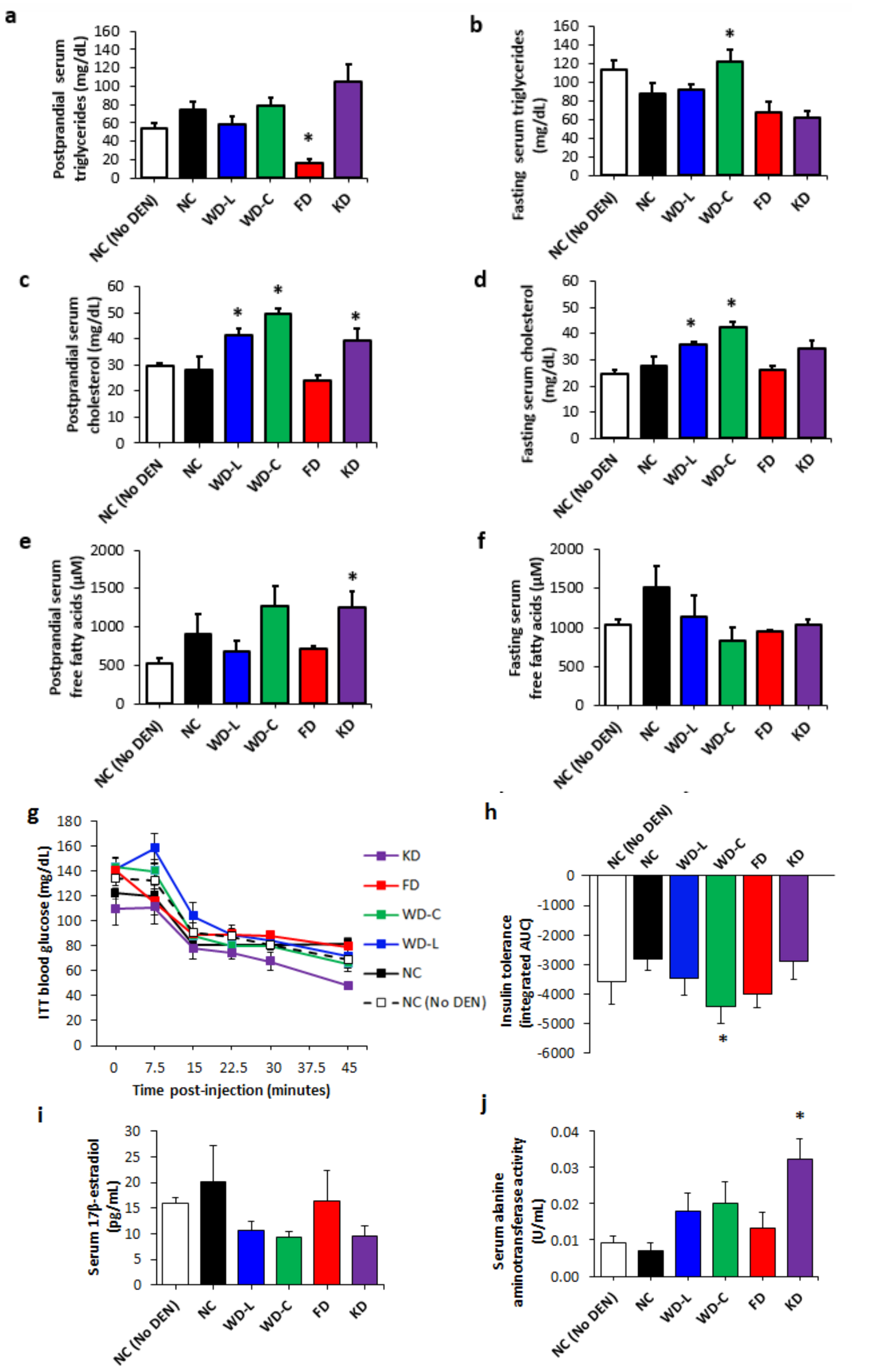

Figure S2. Measurements of serum lipids, estradiol and alanine aminotransferase in mice treated with or without DEN. **(a)** Postprandial and **(b)** fasting serum triglyceride levels, **(c)** postprandial and **(d)** fasting serum triglyceride levels, **(e)** postprandial and **(f)** fasting serum free fatty acid levels in 22-week old mice. **(g)** Blood glucose levels in mice during an insulin tolerance test and **(h)** integrated area under the curve (AUC) in 22-week old mice. Serum **(i)** 17β-estradiol levels in 40-week old mice. **(j)** Serum Alanine Aminotransferase activity in 22-week-old mice. * indicates significant difference from NC, $p < 0.05$. Data are represented as mean ± SEM (n=5-9).

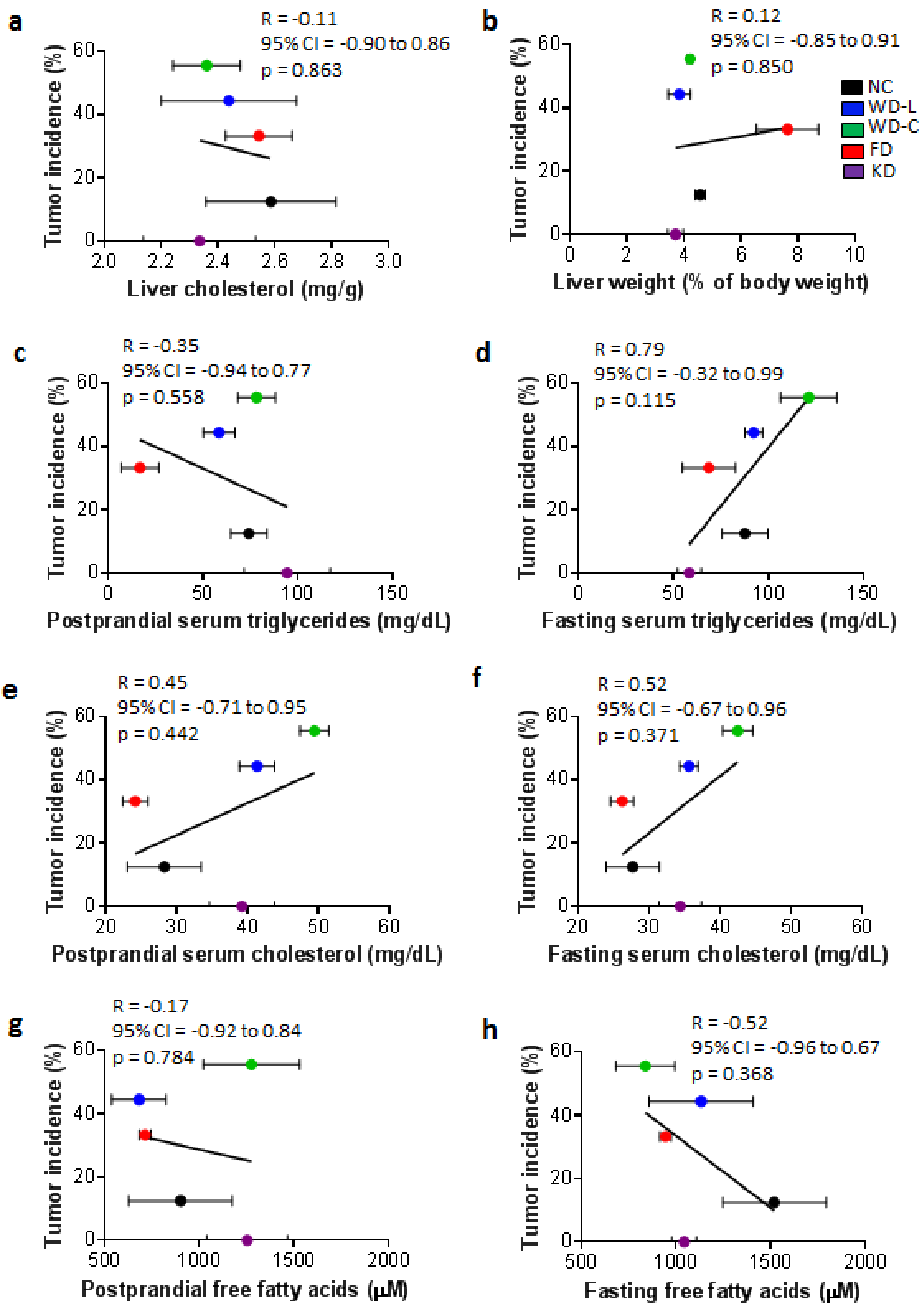

Figure S3. Correlations of metabolic parameters with tumor incidence in DEN-treated female mice. Correlations between tumor incidence and **(a)** liver cholesterol, **(b)** liver weights, **(c)** postprandial and **(d)** fasting serum triglycerides, **(e)** postprandial and **(f)** fasting serum cholesterol, and **(g)** postprandial and **(h)** fasting serum free fatty acids. Linear regression and Pearson's correlation analyses were used to test for correlations with tumor incidence. Data are represented as mean ± SEM (n=5-9).

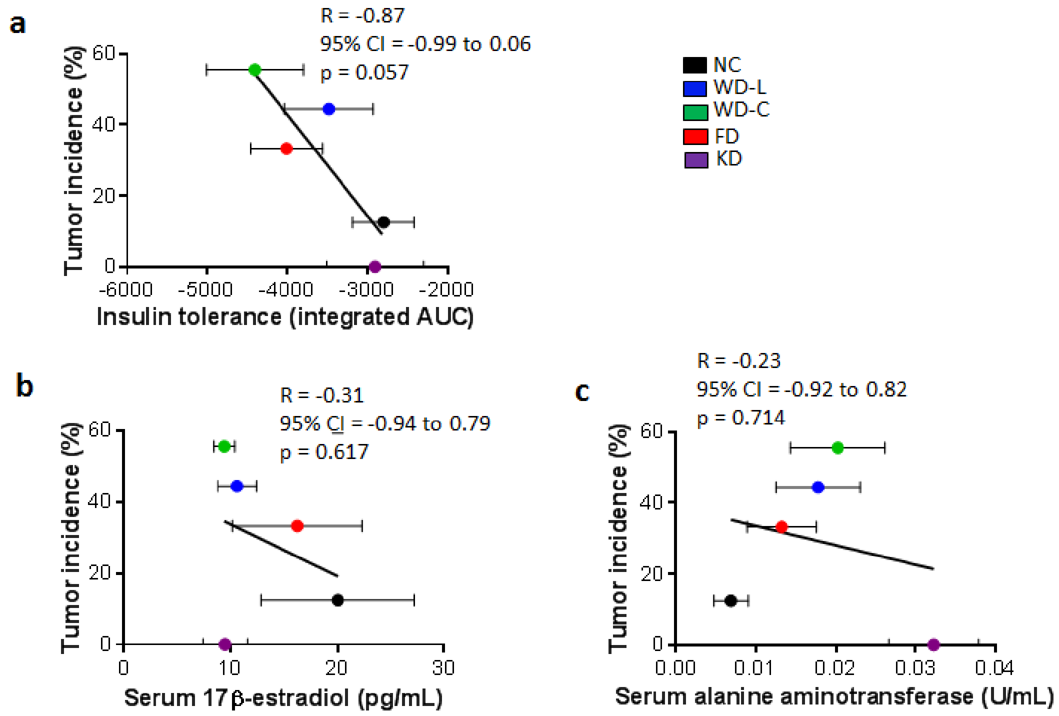

Figure S4. Correlations of insulin tolerance, serum estrogen and serum alanine aminotransferase with tumor incidence in DEN-treated female mice. Correlations between tumor incidence and **(a)** insulin tolerance test integrated AUC, **(b)** serum 17β-estradiol and **(c)** alanine aminotransferase. Linear regression and Pearson's correlation analyses were used to test for correlations with tumor incidence. Data are represented as mean ± SEM (n=5-9).
